# The impact of the co-registration technique and analysis methodology in comparison studies between advanced imaging modalities and whole-mount-histology reference in primary prostate cancer

**DOI:** 10.1038/s41598-021-85028-5

**Published:** 2021-03-12

**Authors:** Constantinos Zamboglou, Maria Kramer, Selina Kiefer, Peter Bronsert, Lara Ceci, August Sigle, Wolfgang Schultze-Seemann, Cordula A. Jilg, Tanja Sprave, Thomas F. Fassbender, Nils H. Nicolay, Juri Ruf, Matthias Benndorf, Anca L. Grosu, Simon K. B. Spohn

**Affiliations:** 1 Department of Radiation Oncology, Faculty of Medicine, Medical Center – University of Freiburg, Freiburg, Germany; 2 Institute for Surgical Pathology, Faculty of Medicine, Medical Center – University of Freiburg, Freiburg, Germany; 3 Department of Urology, Faculty of Medicine, Medical Center – University of Freiburg, Freiburg, Germany; 4 Department of Nuclear Medicine, Faculty of Medicine, Medical Center – University of Freiburg, Freiburg, Germany; 5 Department of Radiology, Faculty of Medicine, Medical Center – University of Freiburg, Freiburg, Germany; 6 German Cancer Consortium (DKTK), Partner Site Freiburg, Freiburg, Germany; 7 Berta-Ottenstein-Programme, Faculty of Medicine – University of Freiburg, Freiburg, Germany; 8 Tumorbank Comprehensive Cancer Center Freiburg, Faculty of Medicine, Medical Center – University of Freiburg, Freiburg, Germany

**Keywords:** Cancer imaging, Prostate cancer, Cancer therapy

## Abstract

Comparison studies using histopathology as standard of reference enable a validation of the diagnostic performance of imaging methods. This study analysed (1) the impact of different image-histopathology co-registration pathways, (2) the impact of the applied data analysis method and (3) intraindividually compared multiparametric magnet resonance tomography (mpMRI) and prostate specific membrane antigen positron emission tomography (PSMA-PET) by using the different approaches. Ten patients with primary PCa who underwent mpMRI and [^18^F]PSMA-1007 PET/CT followed by prostatectomy were prospectively enrolled. We demonstrate that the choice of the intermediate registration step [(1) via ex-vivo CT or (2) mpMRI] does not significantly affect the performance of the registration framework. Comparison of analysis methods revealed that methods using high spatial resolutions e.g. quadrant-based slice-by-slice analysis are beneficial for a differentiated analysis of performance, compared to methods with a lower resolution (segment-based analysis with 6 or 18 segments and lesions-based analysis). Furthermore, PSMA-PET outperformed mpMRI for intraprostatic PCa detection in terms of sensitivity (median %: 83–85 vs. 60–69, *p* < 0.04) with similar specificity (median %: 74–93.8 vs. 100) using both registration pathways. To conclude, the choice of an intermediate registration pathway does not significantly affect registration performance, analysis methods with high spatial resolution are preferable and PSMA-PET outperformed mpMRI in terms of sensitivity in our cohort.

## Introduction

Multiparametric magnetic resonance imaging (mpMRI) is the gold standard for local staging of primary prostate cancer (PCa)^1^. In recent years, positron emission tomography with prostate specific membrane antigen (PSMA)-labelled tracers has emerged as a useful technique for diagnostics and staging of primary and recurrent PCa^2–5^. Accurate identification and delineation of intraprostatic tumor lesions is particularly important for individualized focal therapy approaches such as focal dose escalated radiotherapy (RT)^6^, high intensity focused ultrasound or irreversible electroporation^7^. In order to evaluate and compare performance of mpMRI and PSMA-PET, our team and other workgroups conducted comparison studies using histopathology as standard of reference. Most of these studies demonstrated a superiority of PSMA-PET in terms of sensitivity with similar specificity^2,8–12^, whereas Kesch et al. reported contrary results for [^18^F]PSMA-1007 labelled tracers^10^. Furthermore, studies from Priester et al. and Johnson et al. showed that mpMRI underestimates true tumor volume and misses clinically significant lesions^13,14^. Registration methodology varies between studies and so far, no general applicable workflow has been formulated. However, registration of histological images is challenging, since the prostate deforms non-linearly after prostatectomy, formalin embedding and cutting. Intermediate registration via ex-vivo imaging takes these aspects into account^15–17^. Nevertheless, mismatch susceptibilities are postulated due to discrepancies in depiction of the prostate in CT and MRI images. Additionally, studies employed different spatial resolutions to derive metrics. For instance, approaches include division of each CT-slice into 4 quadrants, division of the prostate into 18 segments or into sextants, voxel-based analysis or were limited to lesion-based analysis^2,10,18–20^.

The current study compared performance for intraprostatic tumor lesion detection of mpMRI with [^18^F]PSMA-1007 PET using intermediate co-registration via ex-vivo CT and ex vivo-MRI as well as different analysis methods. Thereby we aimed to systematically evaluate the impact of the applied co-registration technique and methodology for quantification of spatial overlap in imaging versus histopathology comparison studies in primary PCa patients.

## Material and methods

### Patients

**Figure 1:**
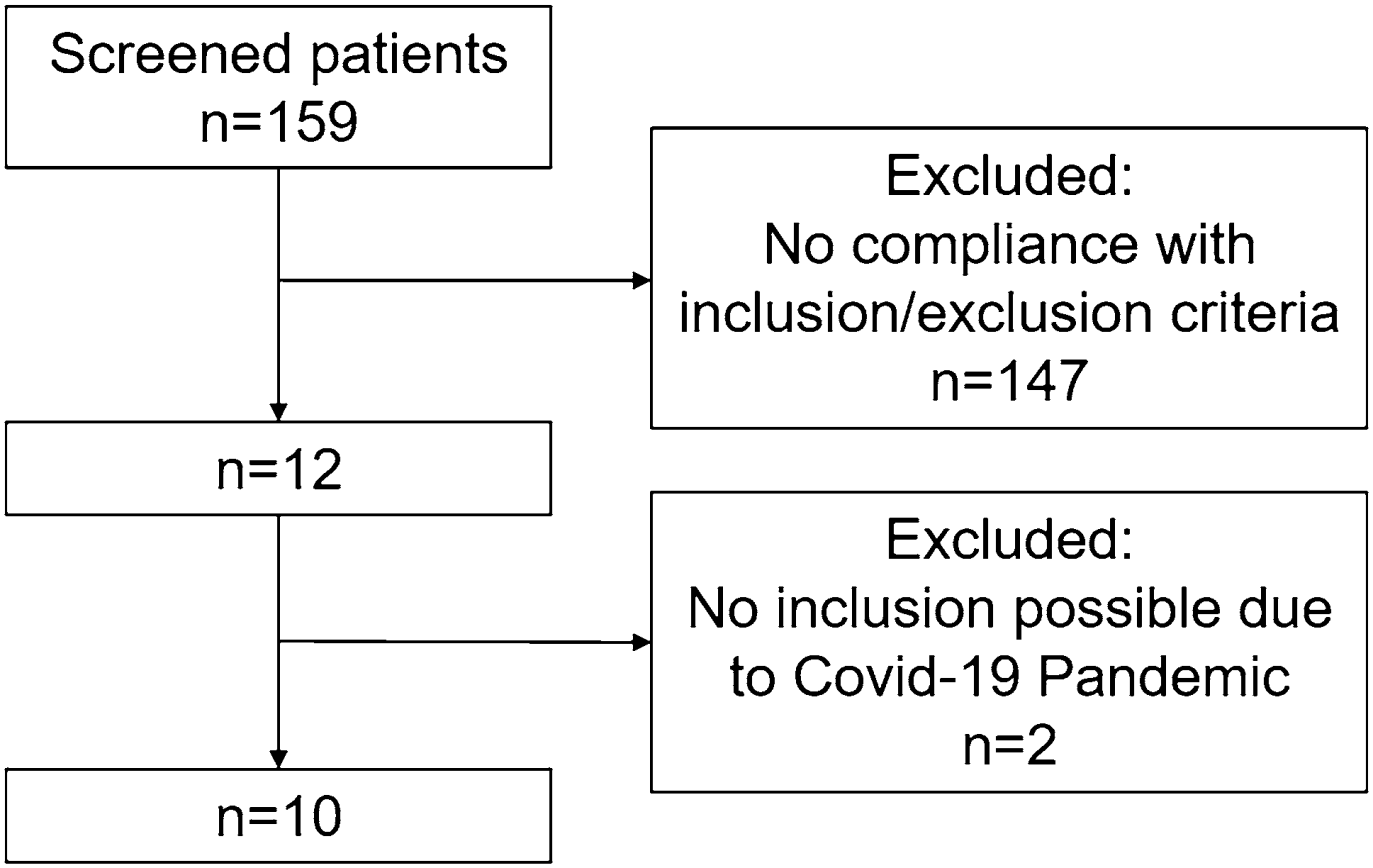
Flow chart for patient inclusion.

Between June 2019 and November 2020, 10 patients were prospectively enrolled. Inclusion criteria were histopathological proven primary PCa, pre-therapeutic [^18^F]1007-PSMA/CT scan, 3Tesla (T) mpMRI and scheduled radical prostatectomy. Exclusion criteria were neoadjuvant androgen deprivation therapy and transurethral prostate resection. Written informed consent was obtained from all patients. See Fig. 1 for the flowchart for patient inclusion. The institutional review board of the Albert-Ludwigs-University Freiburg (Germany) approved the study (No. 476/19).

### MR imaging

In-vivo prostate MRI was performed with clinical 3 T magnets (7 patients with MAGNETOM Trio Tim, 2 patients with MAGNETOM Skyra, 1 patient with MAGNETOM Vida; all Siemens, Germany). For image acquisition a surface phased array coil was combined with the integrated spine array coil, no endorectal coil was used.

In all patients we performed at least biplanar T2 weighted imaging (axial, slice thickness: 2 mm for MAGNETOM Trio Tim, 3 mm for MAGNETOM Skyra and Vida; no interslice gap. Sagittal, slice thickness: 3 mm for all examinations, no interslice gap) and diffusion weighted imaging (low b-value Trio Tim and Vida: 50 s/mm^2^, Skyra: 0 s/mm^2^, high b-value Trio Tim: 800 s/mm^2^, Skyra and Vida: 1000 s/mm^2^). For each patient, a very high b-value image was extrapolated with b = 1400 s/mm^2^ following PI-RADS recommendations. DWI slice thickness was 3 mm in all examinations, no interslice gap was used. Dynamic contrast enhanced images were acquired in the patients examined with Skyra and Vida, slice thickness was 3 mm, no interslice gap was used.

One board certified radiologist (MB) and one board certified radiation oncologist (CZ) with > 6 years’ experience in interpretation of prostate MRI delineated all areas suspicious for significant tumor in the axial T2w sequences (GTV-MRI) in consensus. For delineation T2w images, DWI (including the extrapolated b-value image) and ADC maps were available. Standardized imaging criteria (PI-RADSv2.1) were applied for tumor delineation. Lesions with a PI-RADS category ≥ 3 were considered positive. An experienced reader (CZ) delineated the prostate volume on ex-vivo and in-vivo MRI according to ESTRO-ACROP guidelines^21^.

### PET Imaging

[^18^F]PSMA-1007 had been synthesized according to Cardinale et al.^22^. The mean injected activity of [^18^F]PSMA-1007 was 310 MBq (min–max: 249–370 MBq). Patients underwent a whole-body PET scan starting 2 h after injection. Scans were performed with a 64-slice Vereos PET/CT scanner in all 10 patients (Philips Healthcare, USA). At the time of the PET scan, a contrast-enhanced diagnostic CT scan (120 kVp, 100–400 mAs, dose modulation) was performed for attenuation correction. The uptake of [^18^F]PSMA-1007 was quantified in terms of standardized uptake values (SUV) normalized body weight.

Two radiation oncologists with 6 (CZ) and 2 years (SS) experience in interpretation of PSMA-PET images, respectively, contoured GTV-PET in consensus by applying SUVmin–max 0–10^23^ in Eclipse v15.1 software (Varian Medical Systems, USA). The presence of PCa on PET images was defined as mono- or multifocal uptake greater than adjacent background in more than one slice (GTV-PET). Apart from PET and CT images for anatomical orientation, no additional clinical information was provided. The prostate volume on CT was delineated by an experienced reader (CZ).

### Histopathology and PET/CT image co-registration

**Figure 2:**
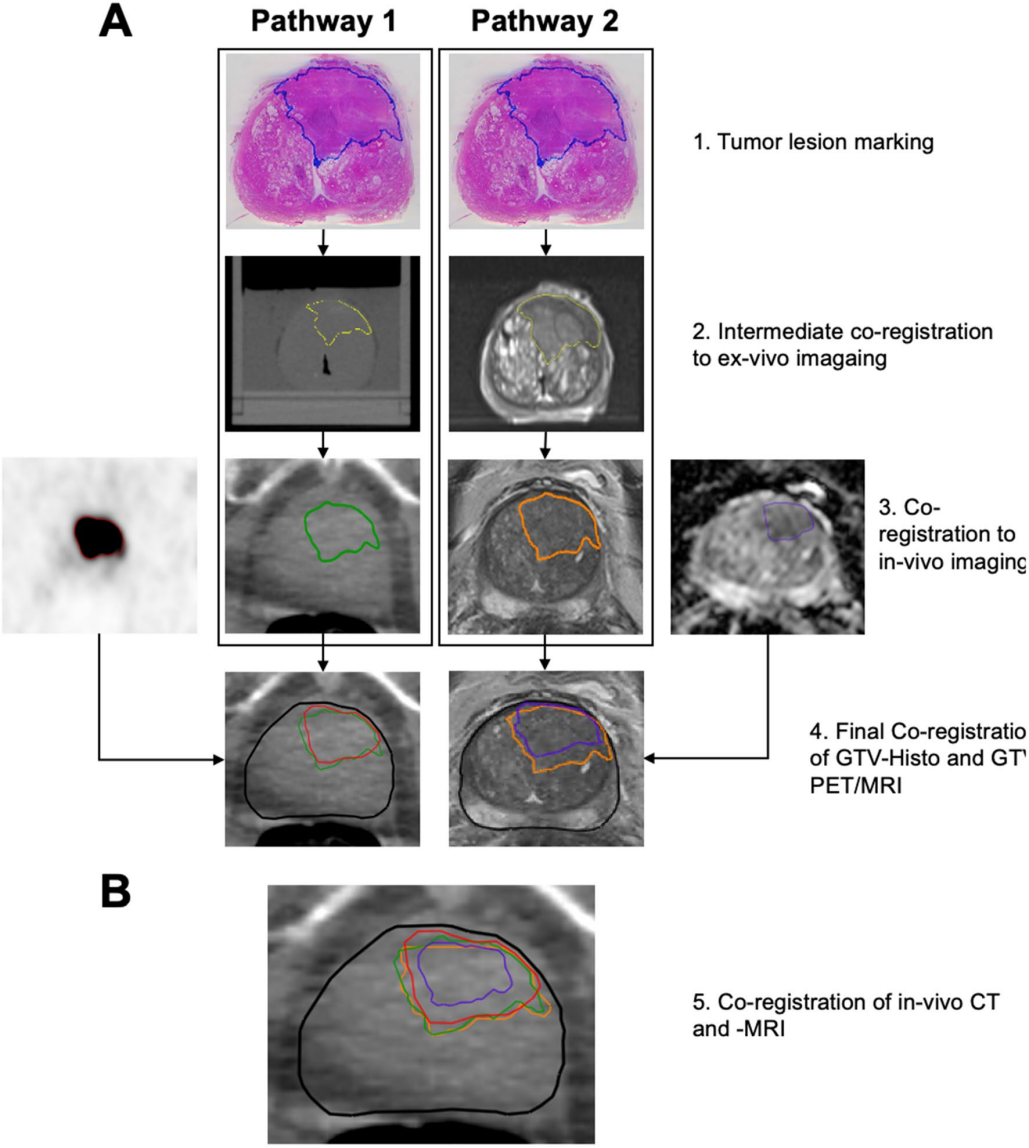
Illustration of the two pathways for co-registration of histopathological slices with in-vivo imaging. (**A**) shows co-registration workflow via intermediate ex-vivo CT (1) and ex-vivo MRI (2) imaging. First row shows H&E stained whole mount prostatectomy specimen with intraprostatic tumor lesions marked in blue. Second row shows intermediate GTV-Histo 1 and GTV-Histo 2 on ex-vivo CT (1) and ex-vivo MRI (2) in yellow (created in MITK Workbench 2015.5.2). Correct localisation of specimen slices was based on the 4 mm grid attached to the side walls of the localizer as well as anatomical landmarks. Third row shows GTV-Histo 1 (green) and GTV-Histo 2 (orange) after final co-registration with in-vivo CT (1) and in-vivo MRI (2). Additionally GTV-PET (red) in diagnostic [^18^F]PSMA-1007-PET/CT and GTV-MRI (purple) in diagnostic MRI (DWI ADC map) are shown. Fourth row shows GTV-Histo 1 (green), GTV-PET (red) and GTV-MRI (purple) in in-vivo CT (1) and in-vivo MRI (2) after final co-registration. (**B**). After manual co-registration of in-vivo CT and in-vivo MRI allowing non-rigid deformation, GTV-Histo 1 (green), GTV-Histo 2 (orange), GTV-MRI (purple) and GTV-PET (red) were superimposed on the corresponding in-vivo-CT image.

PCa lesions in whole mount histopathology specimen served as standard of reference and co-registration was performed similar as previously described^19^. Following formalin fixation, the resected prostate underwent ex-vivo CT (16-channel Brilliance Big Bore, Phillips, Germany, 120 kV and 100 mAs (pixel size x, y, z: 0.3 × 0.3 × 2 mm)) and ex-vivo MRI (MAGNETOM Trio Tim, Siemens Germany, axial T2-weighted images, 2 mm slice thickness) scan in a customized localizer with a 4 mm grid attached to the side walls. A customized cutting device was used to cut step sections every 4 mm to guarantee equal cutting angles between tissue specimen and ex-vivo CT/MRI-slices. The 4 mm grid was visible in the ex-vivo CT and MRI images and enabled a correct localisation for cutting the specimen slices matching the ex-vivo image slices. After paraffin embedding, specimens were cut using a leica microtom. Hematoxyline and eosin staining was performed following routine protocols and PCa lesions were marked by one experienced pathologists (SK). Registration of histology slides to in-vivo imaging was performed via intermediate registration to (1) ex-vivo CT (registration pathway 1) and (2) ex-vivo MRI (registration pathway 2) using MITK software (MITK Workbench 2015.5.2) (see Fig. 2). In this step the PCa contours were digitized and manually transferred to (1) ex-vivo CT imaging and (2) ex-vivo MRI imaging, using the 4 mm grid to match the corresponding histological slides to the (1) CT- and (2) MRI-slides. Furthermore, anatomical landmarks were considered for registration between histopathology specimen and ex-vivo imaging, prioritizing agreement of the prostate capsule contours and additionally considering the urethra and cysts. Automatic interpolation was performed to generate 3D volumes: (1) GTV-Histo 1 and (2) GTV-Histo 2. After transformation to DICOM files using the open source 3D slicer software v4.10.2 (http://www.slicer.org)^24^, ex-vivo CT (1) and ex-vivo MRI (2) images were imported into Eclipse v15.1 software (Varian Medical Systems, USA). Subsequently careful manual co-registration using anatomic landmarks (prostate capsule, prostate shape, urethra, cysts and again the 4 mm grid) of (1) ex-vivo CT (including GTV-Histo 1) and in-vivo CT from diagnostic PSMA-PET/CT, as well as (2) ex-vivo MRI (including GTV-Histo 2) and in-vivo MRI was performed, allowing non-rigid deformation. The accuracy of the registration process was evaluated by visual assessment of the contours in terms of plausible deformations and by calculating the Sörensen-dice coefficient (DSC) for spatial overlap of the prostatic gland in ex-vivo and in-vivo CT (1) and ex-vivo and in-vivo MRI (2). Due to the intermediate registration step, the ex-vivo images comprised the contours of the registered histopathology images. Thus, co-registration of ex-vivo and in-vivo images enabled calculation of DSC of prostatic gland contours of histopathology and diagnostic images and indirectly evaluation of the accuracy of radiology-pathology registration. DSC was calculated by: DSC = 2 │A ∩ B│/(│A│ + │B│).

Hence, careful manual co-registration of corresponding in-vivo CT and in-vivo MRI images was performed, allowing non-rigid deformation. Subsequently, all contours (GTV-Histo, GTV-MRI, GTV-PET) were transferred in the corresponding CT and MRI image, respectively, for further analyses. GTVs were trimmed to the prostatic gland and to the region of the prostatic gland used for histopathological examination.

Hypothetically, derivable co-registration pathways include intermediate co-registration via ex-vivo MR followed by co-registration to in-vivo CT and intermediate co-registration via ex-vivo CT followed by co-registration to in-vivo MR. These approaches were not considered in further analyses, since we expected them to bear more risks for registration mismatches and did not expect them to provide any additional information.

### Statistical analysis

Volumes for GTV-Histo, GTV-PET, GTV-MRI as well as intersection volumes were determined in MITK and Eclipse, respectively.

Sensitivities and specificities for GTV-MRI and GTV-PET based on the histology as reference were calculated for both registration pathways following four different approaches.

1. The prostate in each in-vivo CT slice was divided into 4 equal quadrants. Analysis was performed visually using the GTVs obtained. A median of 62 segments (range 34–94) per patient were analysed^2,19^.
2. The prostate was divided into base, mid and apex and consequently each segment was divided into 6 equal parts (3 × 6), resulting in 18 segments per patient^8^.
3. The prostate was divided into six segments (base right/left, mid right/left and apex right/left)^9^.
4. Lesion based analysis: A lesion was counted as one, as long as the interpolated volumes were coherent. Only index lesions with diameter ≥ 1 cm were included in analysis. Spatially separated volumes were counted as separate lesions.

A voxel-level analysis was performed by calculating DSC for spatial overlap of GTV-Histo 1 with GTV-MRI and GTV-PET in in-vivo CT, as well as of GTV-Histo 2 with GTV-MRI and GTV-PET in in-vivo MRI, respectively.

The quadrant-based analysis approach was used for comparison of performance of MRI and [^18^F]PSMA-1007-PET, since it allowed the most differentiated evaluation.

The statistical analysis was performed on GraphPad Prism v8.4.2 (GraphPad Software, USA). Normal distribution was tested using the Shapiro–Wilk test. For not normally distributed variables, Friedman test and uncorrected Dunn’s test was used for comparison of more than two variables and two-sided Wilcoxon matched-pairs signed rank test was used for comparison of two variables (both at a significance level of 0.05). For normally distributed variables, repeated measures one-way ANOVA with the Geisser-Greenhouse correction and Fisher’s LSD was used for comparison of more than two variables and two-sided paired t test was used for comparison of two variables (both at a significance level of 0.05).

### Ethical approval

All procedures performed in studies involving human participants were in accordance with the ethical standards of the institutional and/or national research committee and with the principles of the 1964 Declaration of Helsinki and its later amendments or comparable ethical standards.

### Informed consent

Informed consent was obtained from all individual participants included in the study.

## Results

### Comparison of registration pathways

Median DSC for spatial overlap of the prostatic gland in ex-vivo and in-vivo CT (1) and ex-vivo and in-vivo MRI (2) was 0.8 (range 0.73–0.89) and 0.85 (range 0.73–0.89), respectively. DSC values were not statistically significantly different (*p* = 0.17).

The absolute volume of GTV-Histo was evaluated in ex-vivo images after intermediate co-registration between histopathology slides and ex-vivo images and subsequent interpolation. Intermediate volumes were not statistically different (*p* > 0.99). After final co-registration median volumes of GTV Histo 1 and 2 were 3.5 ml (IQR 2.2–8.8 ml and 3.7 (IQR 2.0–9.8 ml) and still showed no statistically significant differences (*p* = 0.61) (Fig. 1). Moreover, volume differences between intermediate volumes and final volumes were not statistically significant (*p* > 0.08).

**Figure 3:**
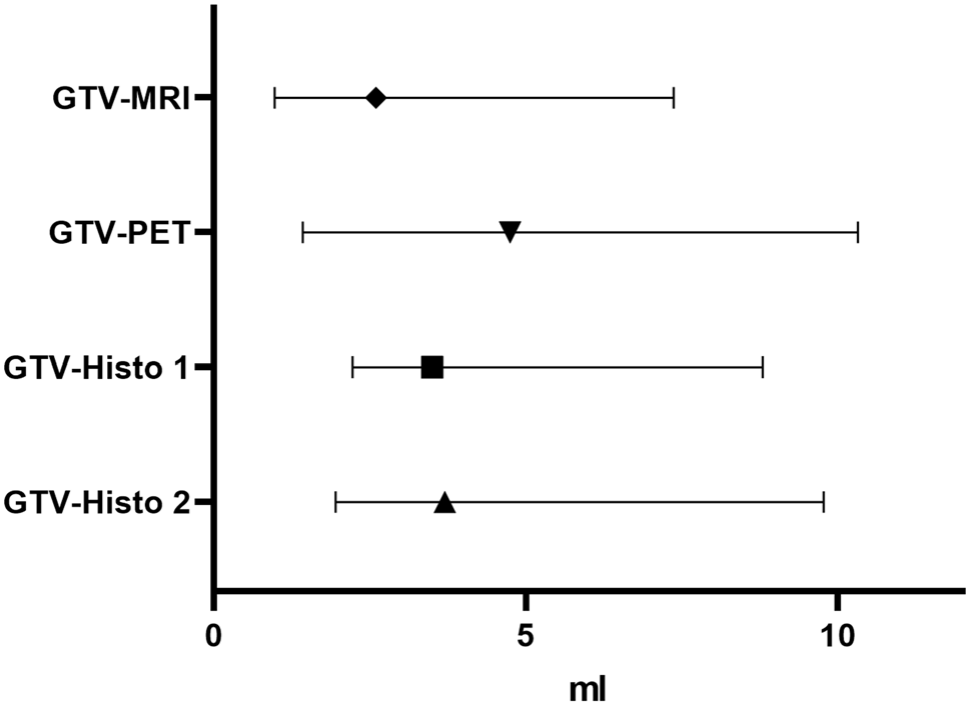
Volumes after final co-registration. Displayed are 3D volumes of histology reference after final co-registration (GTV-Histo 1 and GTV-Histo 2). Additionally volumes contoured in mpMRI (GTV-MRI) and [^18^F]PSMA-1007 PET (GTV-PET) are represented. The median and interquartile ranges over all patients are shown.

After final co-registration, GTV-MRI (median 2.6 ml, IQR 1.0–7.4 ml) was statistically significantly smaller than GTV-Histo 1 and 2 (*p* = 0.015 and 0.019). GTV-PET (median 4.8 ml, IQR 1.4–10.3 ml) was not statistically significantly different to GTV-Histo 1 and 2 (*p* > 0.38) (see Fig. 3). For MRI, analysis of performance using the slice-by-slice and quadrant-based analysis, revealed median sensitivities for registration pathway 1 and 2 of 69% (IQR 61–80%) and 60% (IQR 51–74%) and median specificities of 100% (IQR 92–100%) and 100% (IQR 92–100%), respectively. For [^18^F]PSMA-1007-PET median sensitivities for registration pathway 1 and 2 were 84.5% (IQR 73–96%) and 83.0% (IQR 63–96%) and median specificities of 93.8% (IQR 42–99%) and 74.0% (IQR 47–93%), respectively. See Table 1 for details. Both, sensitivity and specificity showed no statistically significant difference between the two registration pathways for both mpMRI and PET (*p* > 0.05).

**Table 1:** Table 1 shows sensitivities and specificities both histological registration pathways and different analysis approaches for MRI and for PET.

	Method	Quadrant and slice-by-slice		18 Segments		6 Segments	
		Registration pathway 1	Registration pathway 2	Registration pathway 1	Registration pathway 2	Registration pathway 1	Registration pathway 2
MRI	Sensitivity (%)	69.0 (61–80)	60.0 (51–74)	75.0 (65–87)	71.0 (60–83)	75.5 (67–100)	90.0 (67–100)
	Specificity (%)	100.0 (92–100)	100 (92–100)				
PET	Sensitivity (%)	84.5 (73–96)	83.0 (63–96)	91.5 (65–100)	95.5 (70–100)	100.0 (73–100)	100.0 (50–100)
	Specificity (%)	93.8 (42–99)	74 (47–93)				

### Comparison of analysis methods

**Figure 4:**
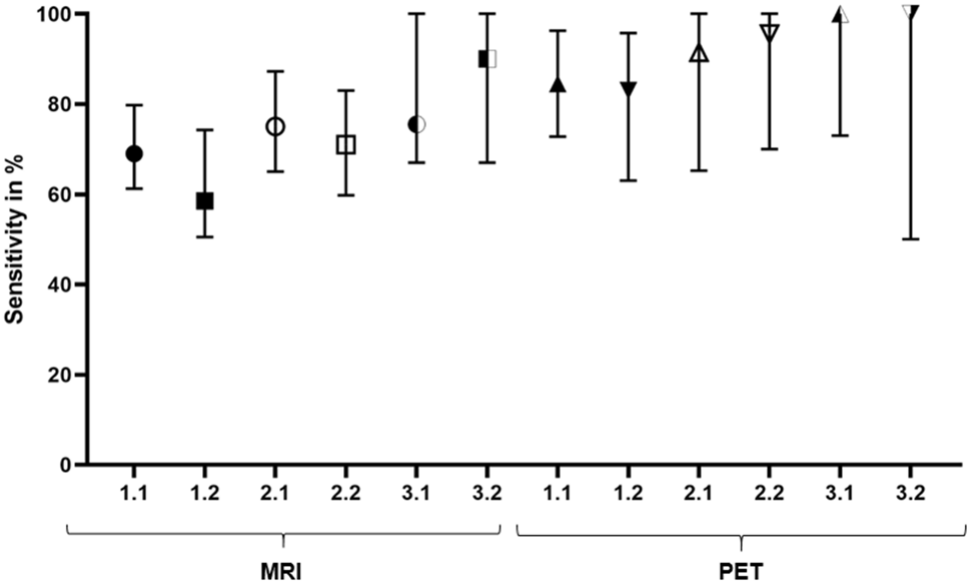
Sensitivity for different analysis methods and registration pathways. The first number indicates the used analysis method (1 = slice-by-slice quadrant based, 2 = 18 segments, 3 = 6 segments) and the second number indicates the used intermediate co-registration pathway: 1 = via ex-vivo CT followed by co-registration of ex-vivo CT and in-vivo CT, 2 = via ex-vivo MRI followed by co-registration of ex-vivo MRI and in-vivo MRI. The median values and interquartile ranges over all patients are shown.

Histology reference revealed 14 index lesions in total. MRI detected all 14 index lesions, PET detected all 14 lesions and one additional lesion (15 lesions in total). See Table 1 and Fig. 4 for sensitivities and specificities for mpMRI and [18^F^]PSMA-1007 using analysis methods 1–3. Sensitivities showed a statistically significant difference for MRI between analysis methods 1 and 3 (*p* = 0.002), as well as 2 and 3 (*p* = 0.023) using registration pathway 2. The remaining comparisons did not show a significant difference for both mpMRI and PET. Furthermore, specificity was only adequately calculable with analysis method 1, since lesions covered all analysed segments when using segment-based analysis approaches in some patients. Therefore, for mpMRI specificitiy was not plausibly determinable for 3 patients when using method 2 and for 4 patients when using method 3. Similarly, PET specificity was not plausibly determinable for 4 patients when using method 2 and for 6 patients when using method 3. Consequently, it was not possible to analyse statistical significance. DSC in in-vivo CT for overlap between GTV Histo 1 and GTV-PET and GTV-MRI was 0.33 (IQR 0.19–0.74) and 0.36 (IQR 0.18–0.54), respectively. DSC in in-vivo MRI for overlap between GTV Histo 2 and GTV-PET and GTV-MRI was 0.32 (IQR 0.19–0.76) and 0.41 (IQR 0.32–0.67), respectively. The Friedman test showed no statistically significant difference of DSCs between registration method 1 and 2 (*p* = 0.27).

### Comparison MRI versus PET

**Figure 5:**
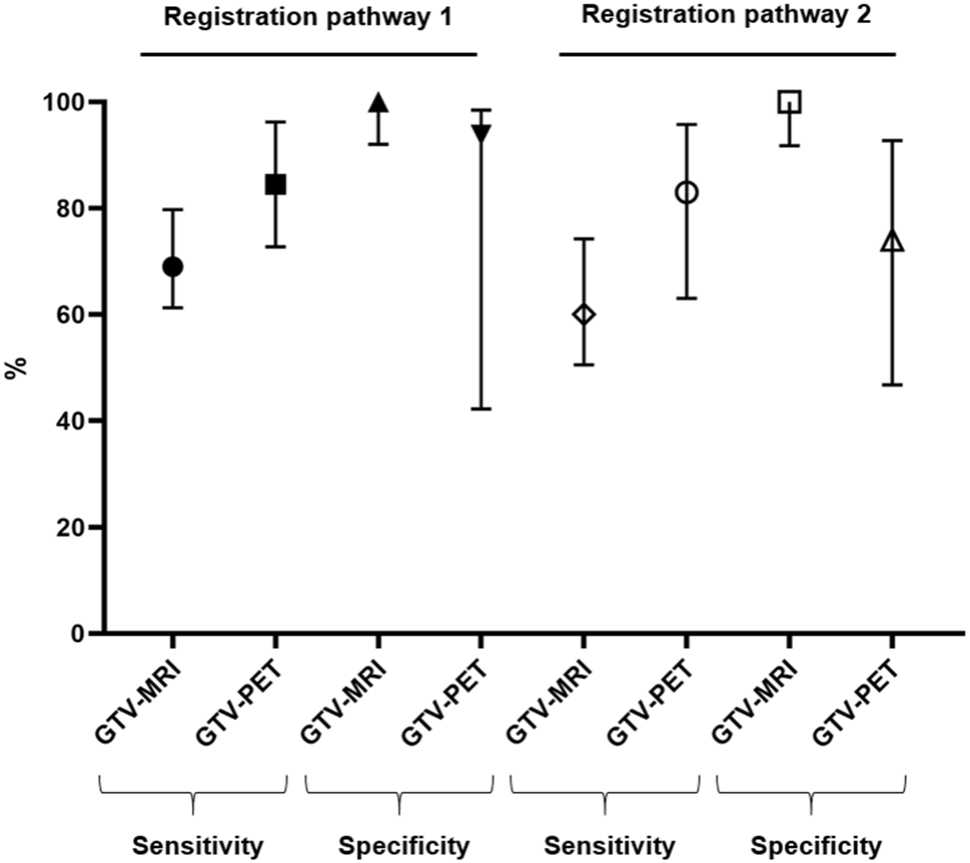
Sensitivity and specificity for mpMRI and PSMA-PET. Displayed are sensitivity and specificity for mpMRI (GTV-MRI) and [^18^F]PSMA-1007 PET (GTV-PET) for registration pathway 1 and 2. The median values and interquartile ranges over all patients are shown.

GTV-MRI was statistically significantly smaller than GTV-PET (*p* = 0.001). See Table 1 for median sensitivity, specificity and IQR for MRI and [^18^F]PSMA-1007 PET, respectively. Using the quadrant based slice-by-slice analysis approach, sensitivity of [^18^F]PSMA-1007 PET was statistically significantly higher for registration pathway 1 (*p* = 0.045) and pathway 2 (*p* = 0.022) (Fig. 5). Specificities were not statistically significantly different for registration pathway 1 (*p* = 0.078), whereas for registration pathway 2 a significant difference could be observed (*p* = 0.02). Evaluation of segment-based analysis approaches revealed a statistically significantly lower sensitivity in mpMRi than in [^18^F]PSMA-1007 PET in registration pathway 2 for analysis method 2 (*p* = 0.02), whereas the remaining comparisons did not show significant difference (*p* > 0.2).

## Discussion

In the setting of individualized biopsy guidance^25^ and focal therapy planning^26,27^, accurate intraprostatic tumor detection and segmentation is pivotal. Sophisticated histopathological comparison studies with high spatial resolution allow drawing more robust conclusions about the imaging’s ability to detect and segment PCa lesions. Comparability of literature is hampered since some studies don’t use histopathology-imaging co-registration and others use variants of registration pathways^2,8–11,15,18,19,28–30^. However, registration of histological images is challenging. Registration results are affected by prostate deformations after prostatectomy, as well as registration mismatches and differences between MRI and CT in depiction of the prostatic gland. Intermediate registration via ex-vivo imaging was introduced to reduce these problems and increase accuracy^15–17^, but different pathways encompass ex-vivo CT and ex-vivo MRI imaging^2,15,19,28–30^. To enhance the comparability of histopathological comparison studies and to analyse whether differences in the used intermediate registration technique significantly affect co-registration results, we evaluated performance for intraprostatic PCa lesion detection of mpMRI and [^18^F]PSMA-1007 PET using intermediate co-registration via ex-vivo CT and ex-vivo-MRI. To the best of our knowledge, this aspect has not been systematically investigated yet. Besides registration of histopathology via intermediate co-registration pathways, the direct registration of digitized pathological slices to radiological images might be a possible alternative^31^. However, prostate deformation after prostatectomy and paraffin embedding impede direct registration via landmark identification. Therefore, we did not perform this approach, anticipating a higher accuracy of co-registration and a true to scale PCa depiction via intermediates.

The intraprostatic tumor volume was not affected by the intermediate co-registration step. Furthermore, sensitivities and specificities did not differ significantly between applied pathways for both, mpMRI and [^18^F]PSMA-1007 PET. Thus, our results suggest that intermediate co-registration via ex-vivo CT and ex-vivo MRI yields comparable results and the choice of the pathway used does not significantly affect registration results. Consequently, these findings enable comparability of previously conducted and future histopathological comparison studies using either registration pathway. Possibly, post prostatectomy transformations of the specimen equally influence both ex-vivo imaging modalities and non-rigid deformation during co-registration process seems sufficient to outweigh or equalize differences in prostate and PCa depiction. Notably, in voxel-based examination DSC was not statistically significantly different between registration pathway 1 or 2. Considering previously reported underestimation of tumor volumes by mpMRI^2,13^, GTV-MRI in our study was statistically significantly smaller than GTV-Histo 1 and GTV-Histo 2. Interestingly, intermediate co-registration via ex-vivo MRI did not result in significantly larger volumes for GTV-Histo compared to intermediate co-registration via ex-vivo CT. This finding indicates that the described underestimation based on histopathological co-registration is not due to methodological bias. In this study we used manual fusion of the prostate in ex-vivo and in-vivo images, since Schiller et al. demonstrated that manual co-registration is more precise and stable than (semi-)automatic methods for CT-based registration in this setting^32^. Accordingly, evaluation of DSC between prostatic gland contours in ex-vivo images (comprising the digitized contours of histopathology images) and in-vivo images revealed a good to excellent overlap for the registration pathway and no significant difference between pathway 1 and pathway 2. Furthermore, volumes of GTV-Histo 1 and GTV-Histo 2 after final co-registration were not significantly different to volumes after intermediate co-registration. These results confirm the quality of the registration pathways and exclude significant methodological uncertainties. Comparison with recent studies evaluating different procedures for co-registration of MRI and histopathology images, indicates that our workflow is similarly accurate, but has potential for improvements^16,17,33^. A deep-learning-based approach presented by Shao et al. is of particular interest in this setting, overcoming disadvantages such as expense in input and processing time^34^.

Another key aspect in the conduct of histopathological comparisons is the analysis method implemented to assign correct positive, correct negative, false positive and false negative PCa detection. Voxel-level comparisons offer the highest spatial resolution for histology/image comparison studies^19^. Evaluation of DSC revealed a positive trend for intermediate co-registration via ex-vivo MRI for evaluation of MRI without being statistically significant. However, the calculated DSCs show poor spatial overlap overall, which is explainable by easy impairment of this voxel-based analysis due to volume alterations, that regularly occur in registration processes. Considering this, the uncertainties of histopathology-image co-registration impede voxel-based analysis and therefore DSC results should be interpreted with caution. Whilst our group previously used a quadrant-based slice-by-slice comparison^2,19,30^, other studies divided the prostate into 18^8^ or 6 segments^9,10,18^. We therefore aimed to evaluate whether different analysis method approaches are equivalent and influence the performance outcome. First, in a lesion-based approach MRI and PET detected all 14 index lesions confirmed by histopathology. PET showed one additional, false positive lesion. Considering the remaining approaches, determined sensitivities for mpMRI and PSMA-PET/CT were lower for the analysis methods with higher spatial resolution for both registration pathways. Surprisingly, sensitivities were statistically significantly different only for MRI in registration pathway 2, suggesting a putative impact on level of significance for more sophisticated analysis methods. Additionally, we experienced difficulties and differences between the analysis approaches when determining specificities. The lower the spatial resolution of the utilized approach, the more likely PCa was detectable in all divided segments. Subsequently, calculation of specificity was not plausible, due to the absence of correct negative segments, although the prostate obviously contained macroscopically healthy areas. Since our results only showed a statistically significant difference of sensitivity between the different analysis methods for MRI using registration pathway 2, these findings somehow limit the significance of an elaborate analysis method. However, we want to remark, that our data demonstrate a clear tendency towards higher statistical metrics for methods with lower spatial resolution, likely originating from the lower numbers of evaluated segments and subsequent less subtle differentiation. Furthermore, we report that comparison of mpMRI and [^18^F]PSMA-1007 PET showed a statistically significant difference between both modalities when using the quadrant-based analysis approach and analysis method 2 (3 × 6 segments), whereas application of the remaining segment based-approaches showed no significant difference in the remaining cases. These results support our conclusion that the use of analysis methods with high spatial resolutions, e.g. quadrant-based slice-by-slice analysis, is beneficial for a differentiated analysis of performance in histopathological comparison studies, particularly for the determination of specificity.

Finally, we conducted a comparison of the performance of [^18^F]PSMA-1007 PET and mpMRI for intraprostatic PCa detection using histopathology as standard of reference based on an elaborate co-registration workflow. Our study shows an excellent performance of [^18^F]PSMA-1007 PET, statistically significantly outperforming mpMRI in terms of sensitivity (85% vs. 69% and 83% vs. 60% *p* < 0.04) with similar specificity (94% vs. 100%, *p* = 0.08 and 74% vs. 100%, *p* = 0.02). The significant difference of specificity in registration pathway 2 is explainable due to the larger volumes of GTV-PET especially in patients with high tumor burden, where small differences in correct negative quadrants exert a great impact. Our results for mpMRI performance are similar to previously reported findings from histopathological comparison studies, which taken together reported a median sensitivity of 61.5% (range 44–86%) and a median specificity of 91% (range 64–98)^2,8–12^. Kesch et al. conducted a intraindividual comparison of [^18^F]PSMA-1007 PET and mpMRI, tending to lower sensitivity (71% vs. 86%) and higher specificity (81% vs. 64%) for [^18^F]PSMA-1007 PET^10^. However, the latter study did not use an equivalent co-registration workflow^10,18^. Furthermore, Kesch et al. additionally performed a near-total-agreement analysis, allowing discrepancy of up to 1 compared region. Considering this analysis, [^18^F]PSMA-1007 PET performed slightly better than mpMRI (sensitivity: 93% vs. 92% and specificity: 94% vs. 83%)^10^. Nevertheless, we are the first to report a superiority of [^18^F]PSMA-1007 PET compared to mpMRI for intraprostatic PCa detection and segmentation in terms of sensitivity using a histopathological-co-registration pathway. Our results underline the value of using [^18^F]PSMA-1007 PET for staging and focal therapy planning of primary PCa.

We acknowledge the limitations of our study. First, our study includes only a low number of patients, but reported results of volumes, sensitivity and specificity are in the range of previously published studies^2,8–10,19^. Secondly, we used different scanners for the acquisition of 3 T mpMRI, but applied imaging protocols were comparable. Notably for acquisition of [^18^F]PSMA-1007 PET images, Vereos scanner was used for all patients (Philips Healthcare, USA), underpinning the state-of-the-art PET image quality. Thirdly, we did not use automatic computer-based methods for co-registration but the used manual approach was validated at least for CT-based co-registration^32^. Lastly, the enrolled patients consisted of intermediate- and high-risk PCA patients scheduled for prostatectomy and therefore the observed results may be only valid for this patient group. However, improvements in diagnostics and therapy planning might have the highest benefit for these patients.

To conclude, our results suggest that performance of co-registration framework for creation of histological 3D models for comparison with diagnostic imaging is not significantly affected by the choice of method (ex-vivo CT or ex-vivo MRI) for intermediate registration. The use of analysis methods with high spatial resolutions, e.g. quadrant-based slice-by-slice analysis, is beneficial for a differentiated analysis of performance in histopathological comparison studies. Furthermore, we plead for the usage of uniform analysis approaches to increase comparability between different studies from different institutions. Comparison of [^18^F]PSMA-1007 PET and mpMRI for intraprostatic PCa detection demonstrated superiority of [^18^F]PSMA-1007 PET compared to mpMRI in terms of sensitivity with similar specificity, underlining its significance for intraprostatic staging and tumor-directed therapies of primary PCa.

## Data Availability

The datasets generated during and/or analysed during the current study are available from the corresponding author on reasonable request.
